# Cricotracheostomy in a Patient With Severe Spinal Cord Injury: Its Usefulness in Trauma Patients Requiring Critical Care

**DOI:** 10.7759/cureus.89993

**Published:** 2025-08-13

**Authors:** Mitsunobu Toyosaki, Toshiharu Nakama, Masahiro Tamashiro, Junichi Sasaki

**Affiliations:** 1 Department of Emergency and Critical Care Medicine, Keio University School of Medicine, Tokyo, JPN; 2 Department of Critical Care Medicine, Yuuai Medical Center, Okinawa, JPN

**Keywords:** head and neck trauma, intensive respiratory care, spinal cord injury, tracheostomy placement, tracheostomy procedure, trauma critical care

## Abstract

Patients with severe spinal cord injury (SCI) generally require prolonged mechanical ventilation, and early tracheotomy offers significant benefits. However, spinal immobilization is necessary, and the neck should not be extended in the acute phase, making appropriate positioning for tracheostomy challenging. Cricotracheostomy is a novel method involving a higher tracheal incision than the conventional approach and is often performed by otolaryngologists in patients with anatomical abnormalities. However, its utility in trauma patients requiring critical care remains unestablished. A male patient in his 60s was admitted to the ED with immobilization and breathing difficulties after falling from a standing position. CT revealed no radiological abnormalities of the cervical vertebrae, while MRI showed high signals in the third and fourth cervical vertebrae (C3-4). The patient was diagnosed with SCI without radiographic abnormalities. Owing to weakened respiration, he was placed on a ventilator. Because he did not undergo surgical cervical stabilization, a cervical collar was maintained. On the third post-trauma day (PTD), a cricotracheostomy was performed to avoid interference with the cervical collar and maintain airway safety, despite the absence of anatomical anomalies. On the 36th PTD, the patient was completely weaned from the ventilator, and vocalization was possible using a speech bulb. This case highlights the potential utility of cricotracheostomy in patients with severe SCI.

## Introduction

In patients with severe spinal cord injury (SCI) at or above the fourth cervical vertebra (C4), respiration is affected, often necessitating prolonged mechanical ventilation. Tracheostomy is indicated for patients who require long-term ventilation. Although the optimal timing for tracheostomy in mechanically ventilated patients remains undefined, early tracheostomy in patients with SCI has been shown to reduce morbidity and mortality and is associated with a decreased length of hospital stay [1-6].

Currently, conventional tracheostomy and percutaneous dilational tracheostomy (PDT) are established tracheotomy techniques. PDT is associated with fewer bleeding complications than conventional tracheostomy and demonstrates similar long-term morbidity [7]. Early tracheostomy, including PDT, in patients with SCI can be performed safely even after anterior cervical spinal stabilization, without increasing the risk of infection or other wound complications [8,9]. Although this position is appropriate for tracheostomy, extension of the neck should be avoided to prevent worsening SCI. Inability to extend the neck may contraindicate PDT [10] and can also make performing conventional tracheotomy challenging in some cases.

Cricotracheostomy involves a higher tracheal incision than the conventional method (usually below the first tracheal cartilage) and is often performed in patients with anatomical abnormalities [11]. Most cricotracheostomies are performed by otolaryngologists, and their utility in trauma patients requiring critical care remains poorly investigated.

We report the case of a patient with severe SCI in whom cricotracheostomy was selected due to interference between the tracheostomy site and the neck collar. The procedure was performed by a surgical board-certified emergency intensive care physician (not an otolaryngologist) and resulted in a favorable outcome.

## Case presentation

A man in his 60s was transported to the ED by ambulance after falling from a standing position; he complained of difficulty moving and shortness of breath. There was no loss of consciousness prior to the injury. The patient had a medical history of diabetes, hypertension, gastroesophageal reflux disease, hepatocellular carcinoma, and an allergy to contrast media.

The primary survey results were as follows: airway: remained patent (normal speech); breathing and ventilation: no signs of tension pneumothorax, massive hemothorax, open pneumothorax, or tracheal or bronchial injuries, although he had weak abdominal breathing, respiratory rate was 16/minute, and oxygen saturation was 96% on room air; circulation: no signs of shock and only weak bleeding from a contusion of the forehead, heart rate was 70 beats per minute, and blood pressure was 133/75 mmHg; sensorium: alert and oriented with a Glasgow Coma Scale score of 15, pupils 2(+)/2(+), the patient could not move his limbs at all; body temperature: 36.3°C.

**Figure 1:**
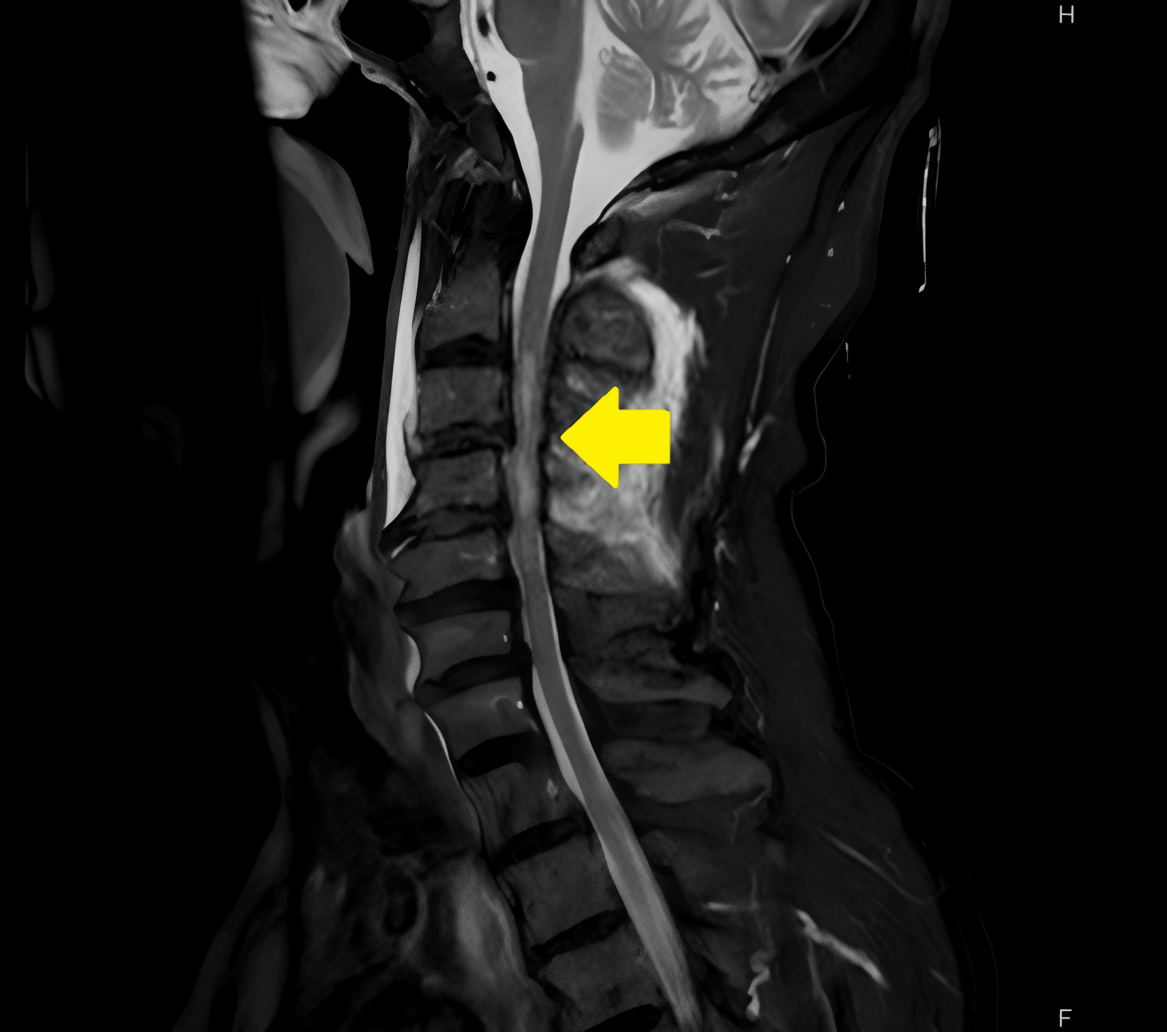
STIR phase of MRI High-intensity signal at T3-4 with no abnormal findings in the cervical vertebrae. STIR, short T1 inversion recovery

**Figure 2:**
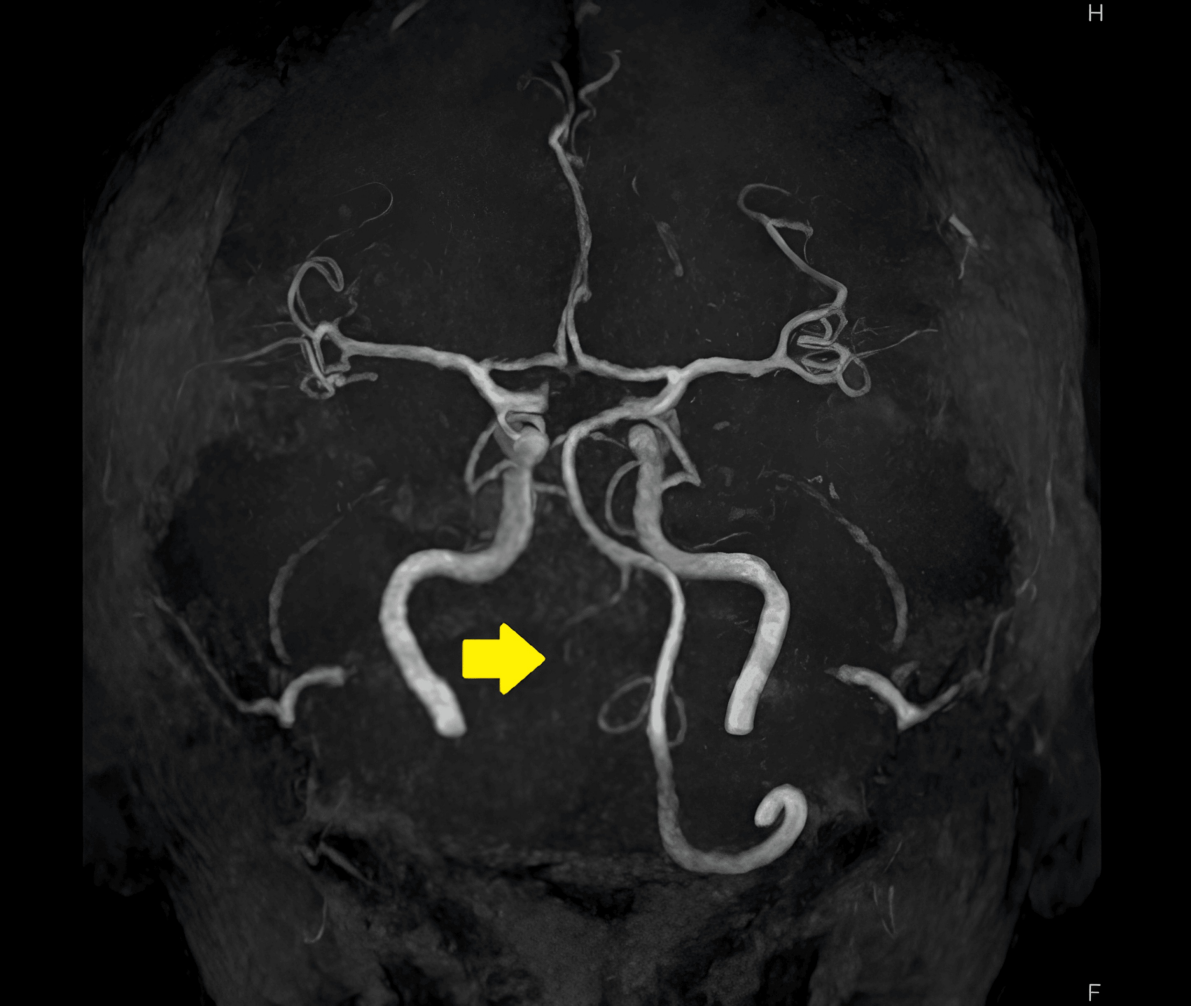
MR angiography The right vertebral artery was not visualized.

In the secondary survey, CT of the pelvic head without intravenous contrast was performed, and no marked injuries were detected. MRI of the vertebrae was performed following CT. High intensity in C3-4 in the short T1 inversion recovery phase was confirmed (Figure 1). Invisibility of the right vertebral artery in MR angiography was suggestive of dissection of the artery (Figure 2).

The manual muscle test results were as follows: trapezius 5/5; deltoids 1/0; biceps 1/0; fingers 1 (thumb only)/0; iliopsoas 0/0; quadriceps femoris 0/0; hamstrings 0/0; tibialis anterior 0/0; extensor hallucis longus 0/0; flexor hallucis longus 0/0; peroneus 0/0; and gastrocnemius 0/0 (Table 1).

**Table 1:** MMT of extremities MMT, manual muscle test

Extremity	Muscles	MMT (R/L)
Upper	Trapezius	5/5
	Deltoids	1/0
	Biceps	1/0
	Fingers	1 (thumb only)/0
Lower	Iliopsoas	0/0
	Quadriceps femoris	0/0
	Hamstring	0/0
	Tibialis anterior	0/0
	Extensor hallucis longus	0/0
	Flexor hallucis longus	0/0
	Peroneus	0/0
	Gastrocnemius	0/0

The upper limb reflexes included biceps tendon reflex (+/+), brachioradial tendon reflex (+/+), Hoffmann’s reflex (-/-), Tromner’s reflex (-/-), and Wartenberg’s reflex (-/-). Lower limb reflexes were patellar tendon reflex (-/-), Achilles tendon reflex (-/-), and Babinski’s reflex (±). Sensory testing revealed light touch sensation at C6/C4 and pinprick sensation at C5/C4. Based on these findings, the patient was diagnosed with spinal cord injury without radiographic abnormalities, classified as Grade A (complete paralysis) on the American Spinal Injury Association impairment scale [12].

Due to weakened respiration, the patient was intubated and placed on mechanical ventilation. Orthopedic surgeons found no cervical spine instability and determined that cervical stabilization surgery was not indicated. Therefore, a cervical collar was maintained for spine protection during the acute phase.

**Figure 3:**
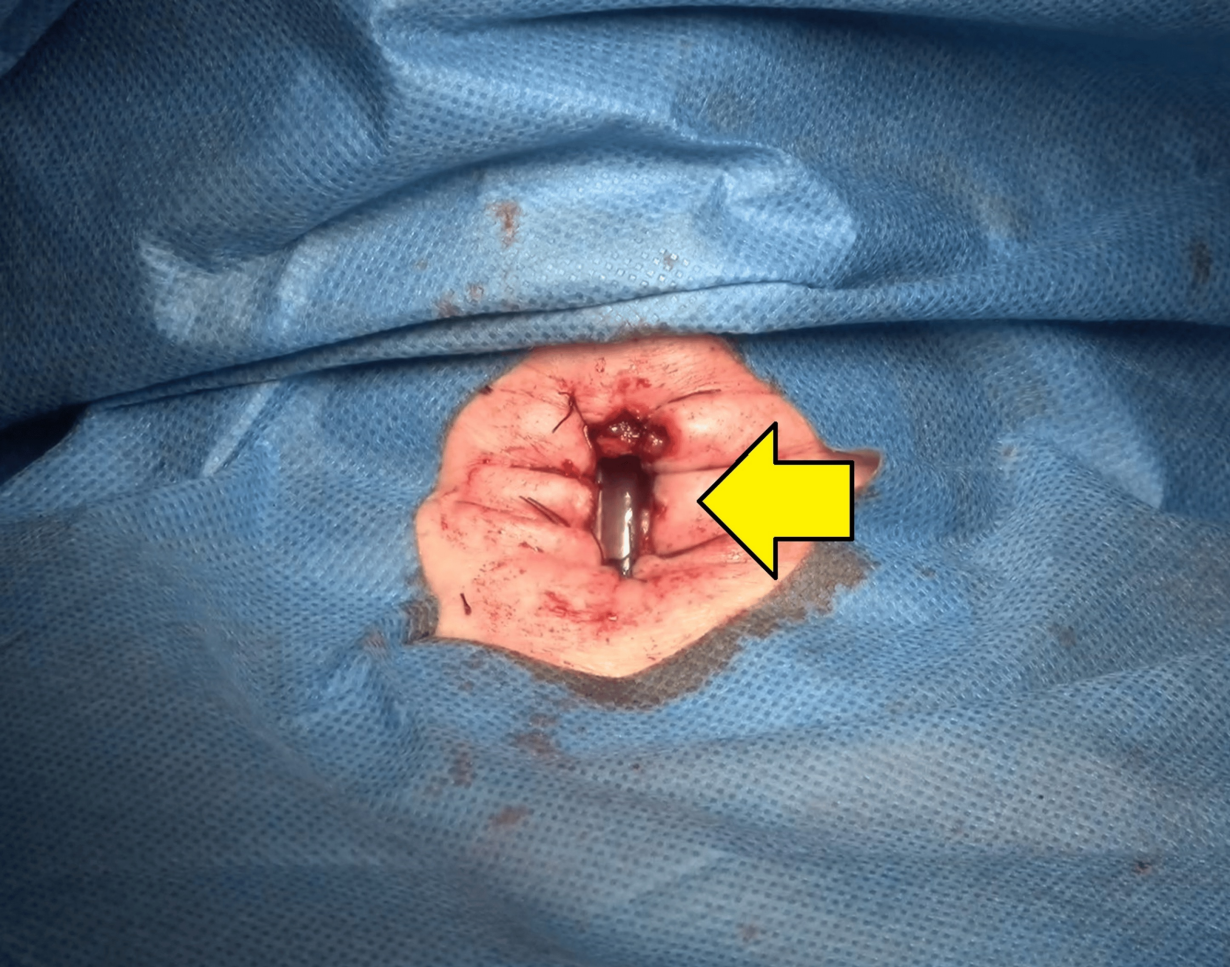
Cricotracheostomy fistula After the removal of the anterior portion of the cricoid cartilage, the fistula was created and secured with eight stitches around it.

On the third post-trauma day (PTD), a surgical board-certified emergency intensive care physician performed a cricotracheostomy, as this method did not interfere with the cervical collar. The procedure followed Kano’s original method [11]; after removal of the anterior cricoid cartilage, eight stitches were placed around the fistula (Figure 3).

**Figure 4:**
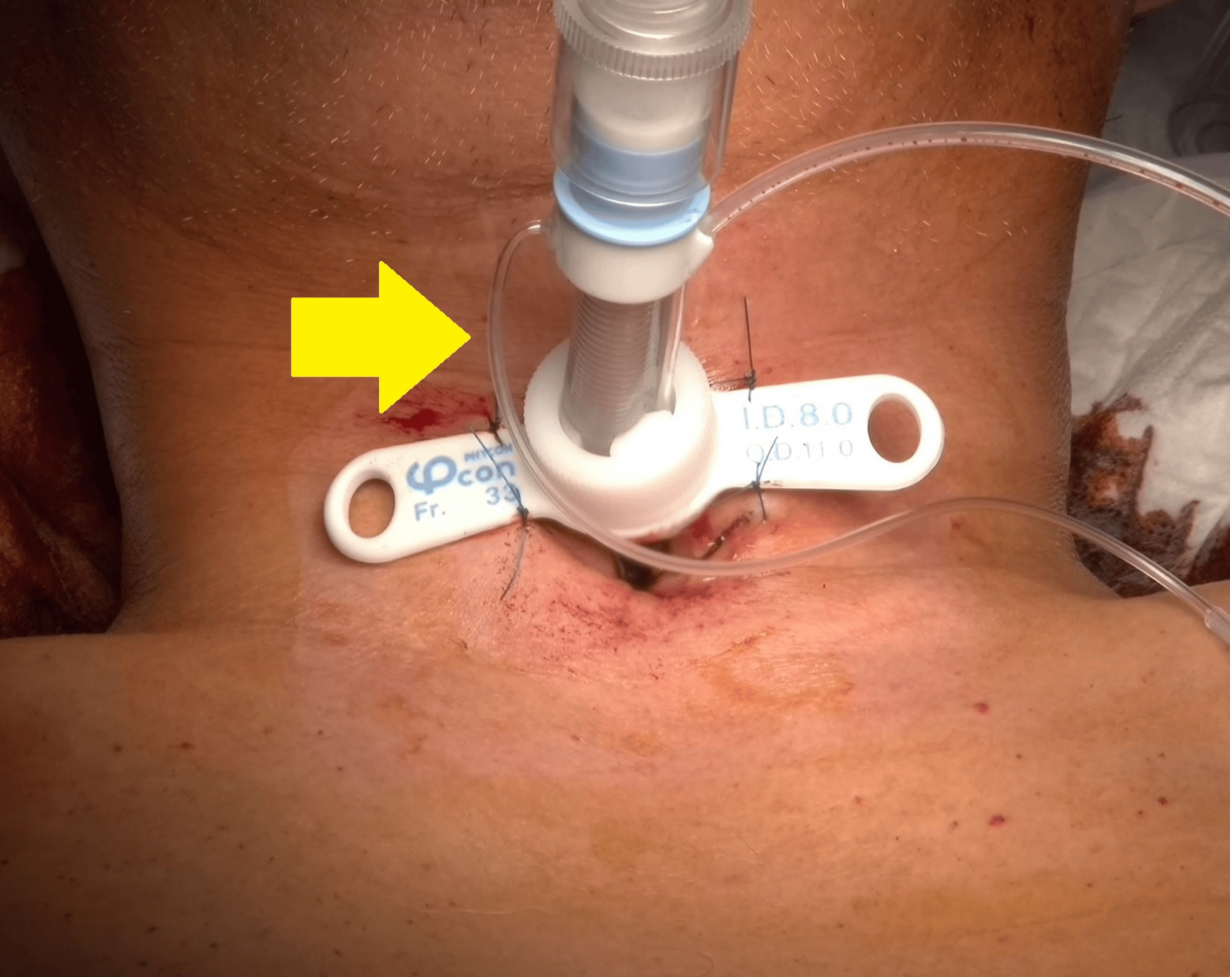
Tracheostomy cannula placement An extended-length tracheostomy cannula was used as the initial cannula to ensure airway safety.

Considering that airway safety was of utmost importance while the cervical collar was in place, an extended-length tracheostomy cannula was used initially (Figure 4).

**Figure 5:**
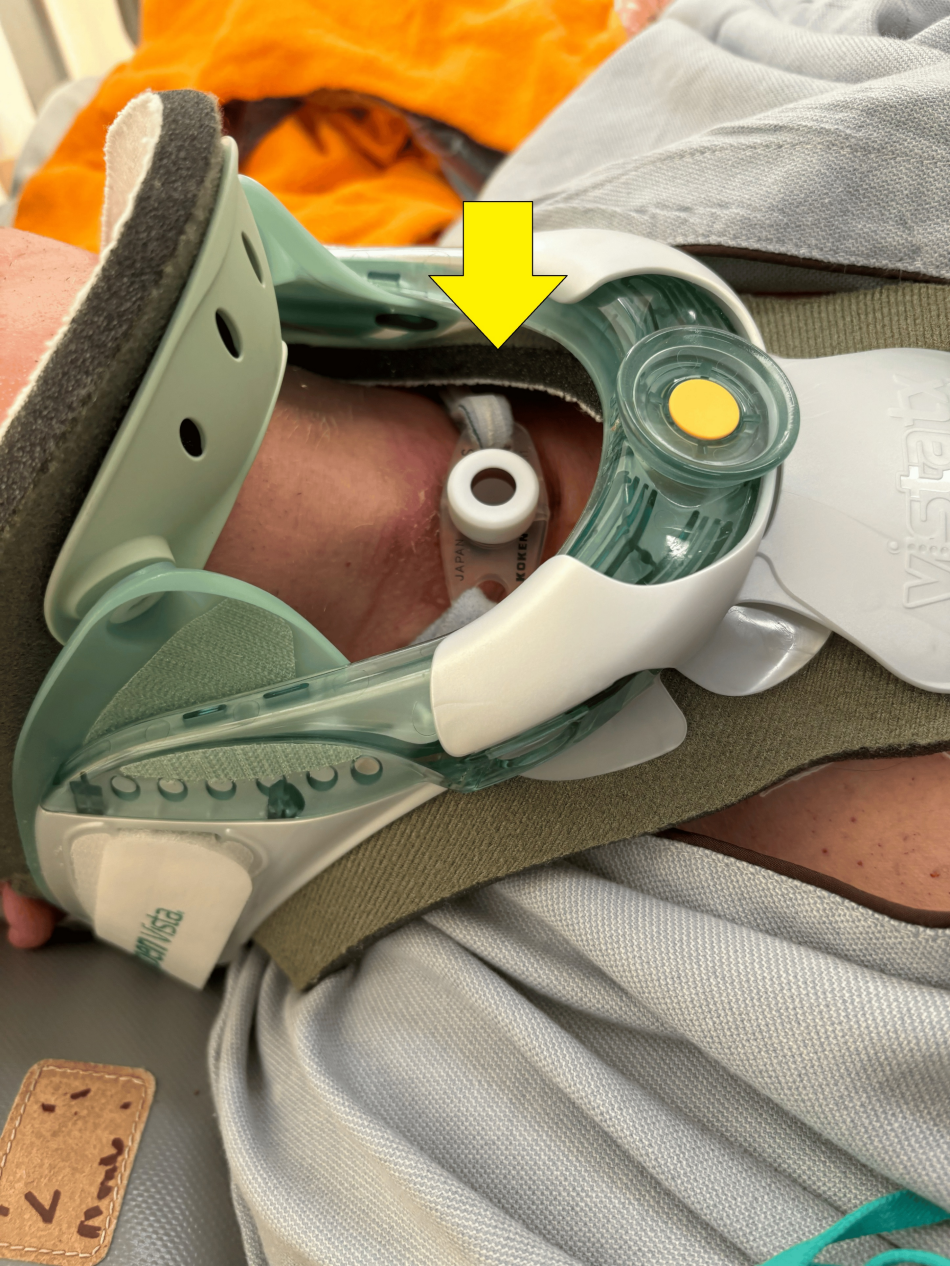
Cannula with speech bulb and cervical collar The cannula did not interfere with the Vista^®^ Cervical Collar (Aspen Medical Products, Irvine, CA, USA).

The patient developed no postoperative complications other than minor surgical site infections. The cannula did not interfere with the collar, and the patient experienced no airway difficulties. On the ninth PTD, the first tracheal cannula exchange was performed without complications. From the 10th PTD onward, the patient was able to consume food orally without aspiration. The patient was completely weaned from mechanical ventilation on the 36th PTD and gradually regained the ability to speak using a speech bulb (Figure 5).

Paralysis below the neck did not improve even after the 50th PTD.

## Discussion

**Figure 6:**
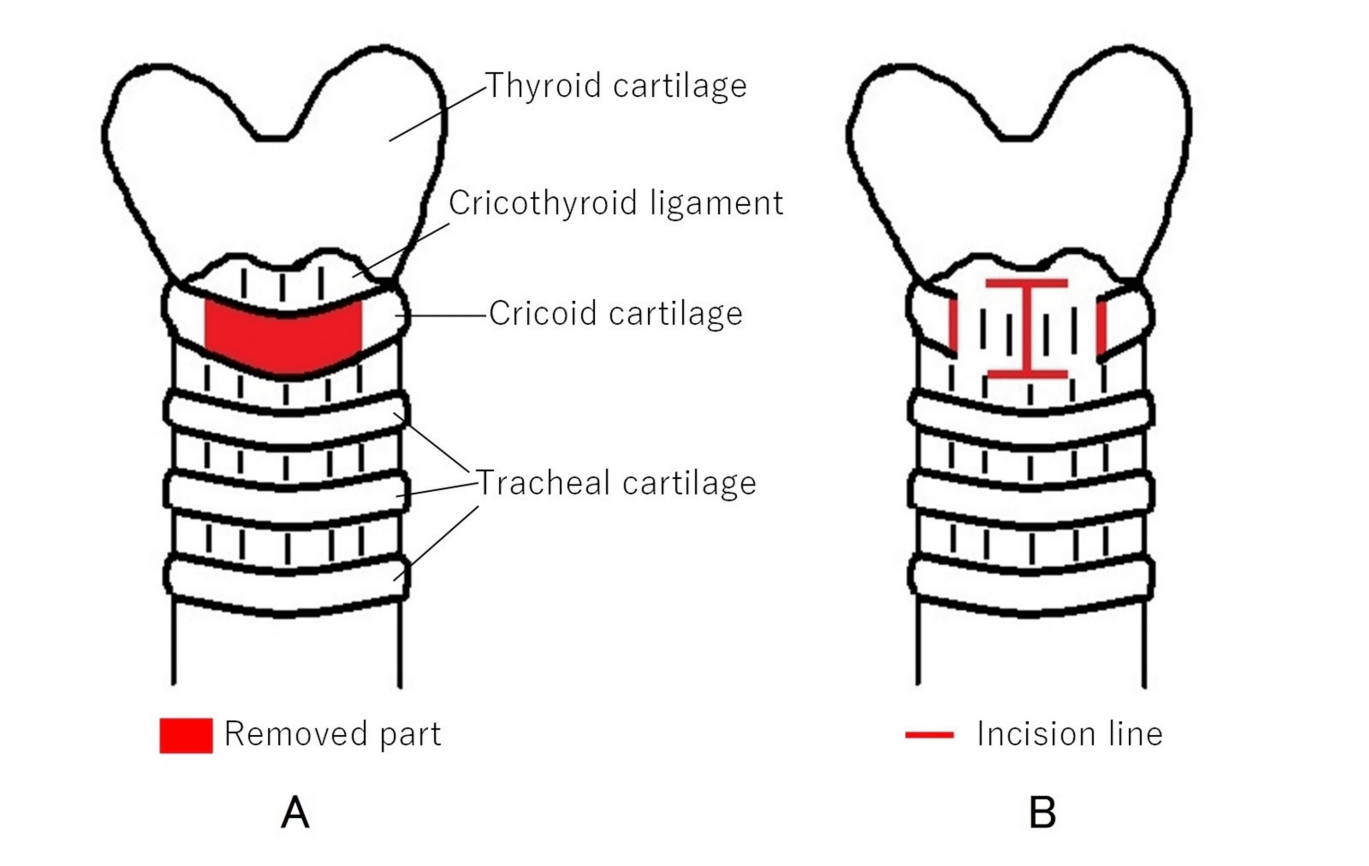
Method of cricotracheostomy (A) The anterior portion of the cricoid cartilage is removed. (B) A horizontal H-shaped incision is made from the cricothyroid ligament to the ligament beneath the cricoid cartilage.

Traditionally, tracheostomy involving the cricoid cartilage has been considered contraindicated due to potential complications such as subglottic stenosis and difficulty with cannula removal [13,14]. Cricotracheostomy involves the removal of the anterior portion of the cricoid cartilage and making an incision in the ligament (Figure 6). This procedure is gradually being adopted by otolaryngologists in cases where conventional tracheostomy is difficult due to anatomical challenges, such as a low larynx, obesity, short neck, aberrant course of the brachiocephalic artery, or the presence of a thyroid tumor [11,15,16].

The apprehension regarding subglottic stenosis [16] and difficulty in cannula removal [15] is not necessarily pronounced, suggesting the inherent safety of the procedure. Cricotracheostomy is a novel technique, and large-scale comparative data remain scarce. However, some studies indicate that it is associated with significantly fewer complications than traditional tracheostomy [17].

Since the cricothyroid muscle, which is involved in speech production, attaches to the lateral side of the cricoid cartilage, cricotracheostomy may affect speech. This method is generally recommended only in cases where tracheal stoma closure is not considered. Nevertheless, a few reported cases have demonstrated restoration of speech after stoma closure [18] or speech availability using a speech bulb [19]. These cases primarily involve intrinsic diseases. Few reports on trauma cases managed by emergency physicians exist, including a solitary case of SCI and two cases of traumatic brain injury [20]. In all instances, cricotracheostomy was chosen due to anatomical obstacles, but none discussed speech outcomes in trauma patients.

In our case, no anatomical hurdles were identified. However, conventional tracheostomy was challenging during the acute phase of SCI because neck extension was not possible. At the conventional tracheostomy incision site, there was a risk of interference with the cervical collar and concerns about cannula stability. After careful consideration, we opted for cricotracheostomy. We observed almost no impact on swallowing, and speech function was maintained. Although further research is necessary, this method is worth considering in SCI cases where preserving speech and swallowing functions is essential.

## Conclusions

We present a case of cricotracheostomy in a patient with severe SCI. The patient had no anatomical abnormalities, and cricotracheostomy was chosen to ensure airway safety without interfering with the cervical collar. The surgery did not affect speech, and no major complications were observed. Cricotracheostomy may be a favorable option not only for patients with anatomical hindrances but also for routine cases of cervical SCI.
